# Phenotype- and age-associated variations in non-specific agglutinins and complement components (C3 and C5a) in camels: Implications for transfusion compatibility and immune function

**DOI:** 10.14202/vetworld.2025.2811-2822

**Published:** 2025-09-23

**Authors:** Yousef M. Alharbi

**Affiliations:** Department of Medical Biosciences, College of Veterinary Medicine, Qassim University, Buraydah, Saudi Arabia

**Keywords:** camel phenotypes, complement C3, complement C5a, innate immunity, non-specific agglutinins, transfusion compatibility

## Abstract

**Background and Aim:**

Blood transfusion in camels is hindered by poorly understood blood group systems, non-specific agglutinins, and a lack of standardized cross-matching protocols. Non-specific agglutinins, primarily immunoglobulin M (IgM), can lead to cross-reactivity, while complement components C3 and C5a impact transfusion outcomes and immune responses. This study aimed to evaluate age- and phenotype-related variations in non-specific agglutinins, C3, and C5a in camels to assess implications for transfusion compatibility and innate immunity.

**Materials and Methods:**

A total of 360 healthy male camels representing three phenotypes (black, yellow, and white) and four age groups (3–5, 5–8, 8–10, and >10 years) were sampled from slaughterhouses in Saudi Arabia. Serum agglutinin titers were determined using hemagglutination assays with heterologous red blood cells (RBCs). Heat inactivation (56°C, 30 min) and sheep RBC (SRBC) adsorption were applied to assess antibody specificity. C3 and C5a concentrations were quantified using an enzyme-linked immunosorbent assay. Statistical analyses employed analysis of variance with Tukey’s *post hoc* test (p<0.05).

**Results:**

Yellow camels exhibited the highest agglutinin titers (up to 1338.4 ± 119.3 against black RBCs), with significant age-related increases. White camels showed the lowest reactivity but demonstrated marked age-related increase in C3 (3.252 ± 0.578 to 4.829 ± 0.983 μg/mL) and C5a (2.776–3.525 μg/mL). Black camels displayed moderate complement levels, peaking in older animals. Heat inactivation and SRBC adsorption substantially reduced titers across all phenotypes, confirming IgM dominance. Age-related increases in agglutinins and complement components indicated immune maturation or cumulative antigen exposure.

**Conclusion:**

Phenotypic and age-related immune differences significantly affect transfusion compatibility in camels. Yellow camels’ high agglutinin activity poses greater transfusion risks, whereas white camels’ lower reactivity and higher complement activity suggest potential as universal donors. Age-adjusted and phenotype-matched transfusion protocols, pre-transfusion heat inactivation, and monitoring C5a in older camels could enhance transfusion safety. This is the first comprehensive study linking camel phenotype and age to complement activation (C3 and C5a), providing a framework for improved transfusion practices and future genomic research into complement-related traits.

## INTRODUCTION

Blood transfusion plays a crucial role in veterinary medicine, particularly in treating conditions such as anemia, trauma, and immune-mediated diseases in camelids [1]. However, the practice of transfusion in camels faces significant challenges due to limited understanding of their blood group systems, the presence of non-specific agglutinins, and a lack of standardized cross-matching protocols. Although blood transfusion offers many advantages, it still poses several risk factors that require further study. Blood transfusions immediately before races are a recent form of doping. In this practice, camel owners withdraw approximately 10 L of blood from one camel and inject it into another before competition to increase oxygen levels, a highly dangerous procedure.

The occurrence of various blood grouping systems in animals is a major concern for modern transfusion medicine. Establishing blood banks could help facilitate safer transfusion practices in camels. Non-specific agglutinins are antibodies that can cause the agglutination of red blood cells (RBCs) without prior sensitization to a specific antigen. These agglutinins are part of the innate immune system and play an important role in the initial defense against pathogens. Understanding variations in non-specific agglutinin levels among different camel phenotypes and age groups has important implications for camel health, immune responses, and potential compatibility issues in blood transfusions or breeding programs. High agglutinin activity can indicate a strong innate immune response, which may be beneficial in resisting infections; however, it can also pose challenges for transfusions and breeding programs, where phenotypic compatibility is crucial.

Isoagglutinin antibodies (immunoglobulin M [IgM] and immunoglobulin G [IgG] isotypes) recognize and bind to antigens. The presence of isoagglutinin antibodies in plasma-derived products can cause serious side effects, such as hemolysis, depending on the type of isoagglutinin present and the blood type of the recipient [2]. It is particularly important to accurately determine isoagglutinin levels to assess the relationship between isoagglutinin exposure and hemolysis. Measurement of the titer is crucial in regulating immune reactions related to transfusion or transplantation [3].

Camels exhibit unique immune system adaptations, including specialized immunoglobulins that may influence complement activation. Their complement system has been less extensively studied than that of other mammals, necessitating the development of tailored methods for measurement and interpretation. Serum complement measurement is a valuable diagnostic and research tool. The complement system is a crucial component of innate immunity, playing a key role in pathogen defense, regulating inflammation, and maintaining tissue homeostasis. Measuring complement in serum samples of camels provides insight into their unique immune adaptations, aiding in disease management and health monitoring. This is particularly valuable for understanding innate immunity and its interplay with adaptive responses, making it a cornerstone of both clinical practice and scientific exploration. Age, sex, and stress can all influence complement activity, and infection, inflammation, and immune disorders may elevate or depress complement levels. Camels adapted to harsh climates may exhibit unique complement system adaptations.

Complement activation can significantly impact transfusion outcomes, influencing recipient immune responses and compatibility [4]. To date, however, no study has comprehensively quantified variations in C3a and C5a among phenotypically distinct camels or examined their impact on cross-matching for transfusion. The degree of complement activation can influence the severity of transfusion reactions. C3 plays a key role in hemolysis, as C3b-mediated opsonization leads to RBC destruction, while C5a functions as a potent inflammatory mediator, amplifying immune responses and contributing to transfusion-related inflammation [4]. The complement system is essential for defending against pathogens and maintaining homeostasis [5]. Elevated C3 levels are associated with increased hemolysis and immune-mediated RBC destruction in transfusion recipients [6]. The development of C5 inhibitors, such as eculizumab, has shown promise in reducing hemolytic complications and improving transfusion safety [7].

Species-specific differences in complement activity highlight the need for customized transfusion protocols in veterinary medicine. Measuring complement components is a valuable tool for predicting transfusion risks and optimizing donor-recipient compatibility [6]. Red cell antigens can be normal or immune-originating antibodies. A low-complex assay is needed for point-of-care testing in low-resource healthcare settings. Blood banks often use hemagglutination (HA) as an alternative method that can be visually observed. HA testing can titrate antibodies and measure their levels in serum. Major cross-matches identify incompatibility between donor RBCs and recipient plasma, while minor cross-matches identify incompatibilities between donor plasma and recipient erythrocytes. Major cross-matches involve detecting agglutinating or hemolytic antibodies in the patient against donor antigens, thereby identifying serological discordance between donor and recipient [1].

Different levels of non-specific agglutinins can be detected in serum samples using RBCs. Agglutination occurs when non-specific agglutinins interact with RBCs, independent of HA caused by microbial antibody–RBC interaction. Specific antibodies to a virus can inhibit virus–RBC interactions but may not affect non-specific agglutinin–RBC interactions, leading to false-negative results in hemagglutination inhibition (HI) assays. Non-specific agglutinins can be detected when diluted serum is mixed with an RBC suspension and agglutination occurs.

Several methods exist to inactivate IgM, including heat inactivation at 63°C and the use of sulfhydryl reagents such as 2-mercaptoethanol or dithiothreitol (DTT). Among these, heat inactivation offers advantages over DTT, including reduced time requirements, no sample dilution, and no need for additional chemical agents, making it a cost-effective option. While heat may denature heat-sensitive proteins, IgG immunoglobulin is relatively more stable at certain temperatures [8].

The endpoints of agglutination reactions are routinely determined by titration, a semi-quantitative method, using conventional test tube technique, column agglutination technology, or solid-phase red cell adherence/HA [9, 10]. These methods require strict standardization to be used effectively, as significant variability exists in the techniques and laboratories performing them [11].

Despite the clinical importance of blood transfusion in camelids, the practice remains hindered by critical knowledge gaps regarding blood group systems, the prevalence and variability of non-specific agglutinins, and the role of complement activation in transfusion compatibility. While previous studies in other livestock species have described the influence of age, breed, and immune status on transfusion outcomes, comparable data for camels are sparse. The limited work that exists on camel transfusion medicine has primarily focused on hematological parameters or serological cross-matching without considering phenotype-specific or age-related differences in immune factors. Moreover, although the complement system, particularly components C3 and C5a, has been identified as a key mediator of transfusion-related hemolysis and inflammation in other mammals, its activity in camels, especially across different phenotypes and age groups, has not been systematically investigated. The absence of such data is particularly problematic in regions where camels are of high economic and cultural value, and where transfusion may be required in emergencies. In addition, non-specific agglutinins are known to cause cross-reactivity in HA assays, but no prior study has quantified their variation across camel phenotypes and ages or evaluated their impact on transfusion safety. This gap in knowledge limits the development of evidence-based transfusion protocols, impedes the establishment of camel blood banks, and may contribute to avoidable adverse transfusion reactions.

This study was designed to address these critical gaps by providing a comprehensive evaluation of non-specific agglutinin activity and complement system components (C3 and C5a) in camels, stratified by phenotype (black, yellow, and white) and age group (3–5, 5–8, 8–10, and >10 years). Specifically, the objectives were to: (1) Quantify agglutinin reactivity against heterologous RBCs in different phenotypes and age groups using HA assays, including pretreatments (heat inactivation and sheep RBC [SRBC] adsorption) to assess antibody class specificity; (2) measure serum C3 and C5a concentrations to evaluate potential phenotype- and age-related differences in complement activity; and (3) correlate these immunological profiles with potential transfusion compatibility risks. By integrating these analyses, the study aims to generate baseline data that can inform phenotype-matched and age-adjusted transfusion protocols, guide blood donor selection, and improve diagnostic accuracy in camel transfusion medicine. Furthermore, the findings may provide a framework for future genetic and immunological studies exploring the basis of phenotypic differences in innate immune function among camels.

## MATERIALS AND METHODS

### Ethical approval

The study was approved by the Animal Ethics Committee of Qassim University (Approval No. 24-07-03) and conducted in compliance with ethical guidelines for animal research and biomedical sample collection. All samples were obtained from camels slaughtered in officially licensed abattoirs under the supervision of local veterinary authorities in Riyadh, Buraydah, Unaizah, and Al-Rass. No live animal experiments or invasive procedures were performed. Blood samples were collected post-slaughter as part of routine meat inspection protocols, thereby exempting the study from prior institutional animal care approval under the Saudi Ministry of Environment, Water and Agriculture guidelines.

### Study period and location

Blood samples were collected from government-regulated slaughterhouses in Riyadh, Buraydah, Unaizah, and Al-Rass during the summer months (June–September 2024). These regions, located between latitudes 24°N and 27°N, are characterized by an arid desert climate, intense solar radiation, and prolonged exposure to dry winds. During the collection period, average daytime temperatures did not exceed 42°C, and relative humidity remained low (20%–30%).

### Animal selection

A total of 360 male camels, representing three phenotypic groups, classified according to Abdallah and Faye [12]; black (Majahem), yellow (Shaele), and white (Wadha), were included in the study. Animals were stratified into four equal age groups (3–5 years, 5–8 years, 8–10 years, and >10 years) based on dentition patterns. Phenotype selection was guided by prevalence in the study areas and the potential for differences in blood characteristics relevant to transfusion compatibility. Age grouping aimed to assess variations in complement activity and non-specific agglutinin levels between younger and older camels.

### Sample classification and collection

The sample size was determined using power analysis (G*Power 3.1) based on preliminary pilot data (n = 30), with an effect size of 0.3, α = 0.05, and 80% power, ensuring sufficient statistical sensitivity to detect phenotype-related differences. All camels were clinically healthy as determined by pre-slaughter inspection, and those exhibiting signs of disease or parasitic infection were excluded from the study.

Blood samples (10 mL) were aseptically collected from the jugular vein immediately post-slaughter, labeled, and transported on ice to the laboratory. The collected volume was based on requirements for subsequent laboratory assays. Humane handling was ensured by trained personnel, minimizing stress. Standardized collection and processing protocols were followed to maintain sample integrity and ensure data validity.

For serum preparation, clotted samples were incubated at 37°C for 20 min, allowed to stand at room temperature (23°C) for ~2 h, and centrifuged until clear. Serum was aliquoted and stored at −79°C in a deep-freezing cabinet.

### Preparation of 1.0% SRBCs for agglutinin adsorption

Fresh sheep blood was obtained from the veterinary hospital and stored in sterile Alsever’s solution (50 mL, pH 7.2) containing citric acid (0.055%), sodium citrate (0.8%), sodium chloride (0.42%), and glucose (2.05%). The suspension was mixed gently by inversion. Sheep RBCs (SRBCs) were washed three times in pyrogen-free, sterile cold PBS containing 0.5% BSA and centrifuged at 600–1,006 × *g*, for 10 min. Final packed RBCs were adjusted to a concentration of 1.5 × 10^8^ cells/mL using a Neubauer chamber. A 1.0% (v/v) SRBC suspension was prepared fresh before use, stored at 4°C, and discarded after 24 h.

### Heat inactivation of serum

Aliquoted serum samples were heat-inactivated at 56°C for 30 min [9] without mixing, then cooled to 23°C. Plasma from different phenotypes was serially diluted in PBS using doubling dilutions, with each successive dilution halving the concentration of the previous sample.

### Cross-matching and direct agglutination assay

Isoagglutinin activity was assessed by direct HA assays using 1% RBC suspensions and serially diluted serum [9, 10]. Microtiter V-bottom plates were used, with serum dilutions of 1:2, 1:4, 1:8, 1:16, and 1:32 tested against heterologous RBCs. Plates were incubated at 23°C or 37°C for 15–30 min, and agglutination was visually assessed.

The endpoint titer was defined as the highest serum dilution with visible agglutination. Tests were performed in duplicate or triplicate, with readings recorded at 30 min, 2 h, and 4 h. Hemolysis scores were recorded (4, 3, 2, 1, trace, 0), and total scores represented antibody strength (e.g., 35 + 3 + 1 = 39). Isoagglutinin titers were visualized by tilting the plate at 45°–60°.

### Measurement of complement components (C5a and C3)

C5a and C3 concentrations were quantified using sandwich enzyme-linked immunosorbent assay kits (Catalog Nos. SL0065Cm, SL0047Cm; SunLong Biotech, China), stored at 2°C–8°C until use. The assay ranges were 16–1,000 pg/mL for C5a and 45–5,000 pg/mL for C3, with sensitivities of 3.2 pg/mL and 8 pg/mL, respectively.

**Figure 1:**
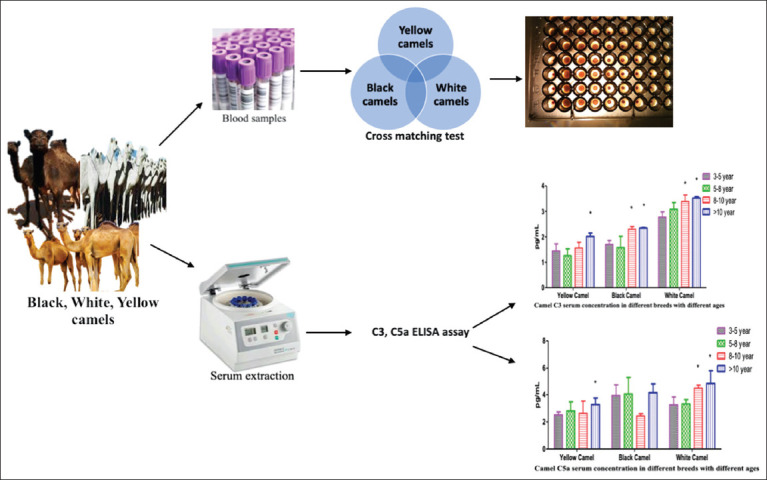
Flowchart of the sample processing.

Technicians blinded to phenotype and age handled all samples. Samples were randomized across plates to minimize batch effects. Intra-assay coefficient of variation (CV) was maintained below 10% and inter-assay CV below 12%. Standard curves (R^2^ > 0.98) were generated for each run. Samples outside the linear range were retested at appropriate dilutions. Optical densities were read at 450 nm and analyzed using log–log regression in GraphPad Prism 9.0. Skewed data were log^10^-transformed, and back-transformed values are reported (Figure 1).

### Statistical analysis

Data are expressed as means ± standard error. One-way analysis of variance was performed for each parameter using SAS 20.0 (SAS Institute, USA). Data normality was evaluated with the Shapiro–Wilk test and Q–Q plots. Log transformation was applied to non-normal data; if assumptions were still violated, Kruskal–Wallis tests were used. Variance homogeneity was assessed with Levene’s test. Tukey’s Honestly Significant Difference test was applied for *post hoc* pairwise comparisons within phenotype and age categories.

## RESULTS

### Non-specific agglutinin reactivity by phenotype and age

Table 1 summarizes the non-specific agglutinin dilution endpoints of sera from yellow, black, and white camels tested against RBCs of different phenotypes, categorized by four age groups (3–5, 5–8, 8–10, and >10 years).

**Table 1:** Non-specific agglutinin dilution endpoint of serum of camel phenotypes against RBC of different camel phenotypes.

Age of camel	Serum of yellow + black RBC	Serum of yellow + white RBC	Serum of black + RBC of yellow	Serum of black + white RBC	Serum of white + RBC of yellow	Serum of white + RBC of black	p-value
3–5 years	216.6 ± 8.4^a^	115.2 ± 22.1^a^	28.4 ± 7.3^b^	28.4 ± 6.7^b^	55.9 ± 11.0^b^	8.0 ± 2.8^b^	0.000
5–8 years	208.4 ± 2.9^a^	102.9 ± 18.6^a^	115.5 ± 22.3^a^	54.8 ± 11.2^b^	56.3 ± 9.4^b^	16.1 ± 3.7^c^	0.00
8–10 years	665.6 ± 7.6^b^	1446.1 ± 102.1^a^	28.6 ± 4.9^c^	53.4 ± 0.2^c^	111.6 ± 33.2^d^	64.6 ± 1.3^c^	0.000
>10 years	1338.4 ± 119.3^a^	1374.4 ± 99.3^a^	196.4 ± 38.9^b^	112.6 ± 14.8^b^	115.5 ± 28.4^b^	64.2 ± 14.9^b^	0.000

Values are expressed as means ± standard error of the mean. Values with different superscript letters (e.g., a, b, and c) within the same row are significantly different (Tukey’s Honestly Significant Difference test, p < 0.05). “a” represents the highest mean value in each row. Groups that share the same letter are not significantly different. RBC: Red blood cells

Yellow camels consistently showed significantly higher agglutinin titers than black and white camels across most age groups. For example, yellow camels aged >10 years had titers of 1338.4 ± 119.3 against black RBCs, approximately 6.2 times higher than the titers in 3–5-year-old yellow camels (216.6 ± 58.4; p < 0.001).

White camels exhibited the lowest reactivity overall; however, their titers against black RBCs rose markedly with age, from 8.0 ± 2.8 (3–5 years) to 64.2 ± 14.9 (>10 years), representing an 8-fold increase (p < 0.001).

Black camels displayed moderate agglutination against yellow RBCs, with titers peaking at 196.4 ± 38.9 in the >10-year group.

Age was a strong determinant of agglutination strength, especially in yellow camels. For instance, titers against black RBCs increased from 216.6 ± 58.4 (3–5 years) to 2048 (>10 years), while titers against white RBCs rose from 128 to 1374.4 ± 99.3 over the same age span. Black camels also showed some age-related increases, but the trend was less consistent, while white camels demonstrated a gradual rise in titers against yellow and black RBCs with advancing age.

### Implications for transfusion compatibility

These phenotypic differences in agglutinin reactivity may have both biological and clinical relevance. The strong reactivity of yellow camel serum suggests a higher risk for adverse reactions in cross-phenotype transfusions, necessitating careful donor-recipient matching. In contrast, the weaker reactivity of white camel serum suggests broader compatibility potential.

The age-related increases in agglutinin titers highlight the dynamic nature of the camel immune system. For diagnostic purposes, both phenotype and age should be considered when interpreting serological test results to avoid misinterpretation or false positives, particularly in yellow camels. Mitigation strategies such as pre-adsorption with SRBCs or heat treatment may help reduce non-specific reactivity.

Future research should investigate the molecular identity of these agglutinins (e.g., IgM vs. IgG) and their clinical implications, including possible roles in hemolytic disease in mixed-phenotype breeding programs.

### Effect of serum treatments on agglutinin titers

Table 2 presents the dilution endpoints of non-specific agglutinins under different conditions: untreated, heated at 56°C for 30 min, and incubated with SRBCs. Data are grouped by phenotype and age.

**Table 2:** Non-specific agglutinin dilution endpoint of serum of camel phenotypes (white, yellow, and black) and after heating at 56°C for 30 min or incubation with SRBC and against RBC of different phenotypes.

Phenotypic serum	Phenotypic RBCs	3–5 years	5–8 years	8–10 years	>10 years	p-value
Serum of yellow	+RBCs of the black	216.6^c^ ± 58.4	208.4^c^ ± 32.9	665.6^b^ ± 27.6	1338.4^a^ ± 119.3	0.000
Pre-incubation with SRBC		6.4 ± 2.1	6.4 ± 2.1	7.2 ± 3.4	7.2 ± 3.4	NS
Heating at 56°C for 30 min		1.6 ± 0.4	1.8 ± 0.3	2.0 ± 0.4	1.6 ± 0.4	NS
Serum of yellow	+RBCs of white	115.2^b^ ± 22.1	102.8^b^ ± 18.6	1446.1^a^ ± 102.1	1338.4^a^ ± 99.3	0.000
Pre-incubation with SRBC		6.4 ± 3.9	7.2 ± 2.1	12.3 ± 4.1	14.4 ± 4.1	NS
Heating at 56°C for 30 min		0. 1 ± 0.0	0.1 ± 0.0	1.8 ± 0.6	1.6 ± 0.9	NS
Serum of the black	+RBCs of yellow	28.4^b^ ± 7.3	115.5^a^ ± 22.3	28.6^b^ ± 4.9	196.4^a^ ± 38.9	0.000
Pre-incubation with SRBC		3.8 ± 1.6	3.6 ± 1.9	3.8 ± 1.6	12.6 ± 3.2	NS
Heating at 56°C for 30 min		3.8 ± 1.6	3.8 ± 1.6	3.6 ± 1.9	3.6 ± 1.9	NS
Serum of the black	+RBC of white	28.4^b^ ± 6.7	54.8^b^ ± 11.2	53.4^b^ ± 9.2	112.6^a^ ± 14.8	0.00
Pre-incubation with SRBC		6.4 ± 3.9	7.2 ± 2.1	12.7 ± 4.2	14.4 ± 4.8	NS
Heating at 56°C for 30 min		1.8 ± 0.6	1.8 ± 0.6	1.6 ± 2.9	2.0 ± 0.8	NS
Serum of white	+RBC of yellow	55.9^b^ ± 11.0	56.3^b^ ± 9.4	111.6^a^ ± 33.2	115.5^a^ ± 28.4	0.000
Pre-incubation with SRBC		3.6 ± 1.6	3.6 ± 1.5	3.8 ± 1.4	3.8 ± 1.4	NS
Heating at 56°C for 30 min		0.1^b^ ± 0.0	1.4^b^ ± 0.3	3.6^b^ ± 1.3	3.8^a^ ± 1.2	0.000
Serum of white	+RBC of the black	8.0^b^ ± 2.8	16.1^b^ ± 3.7	64.6^a^ ± 11.3	64.2^a^ ± 14.9	0.000
Pre-incubation with SRBC		3.8 ± 0.9	2.16 ± 0.3	3.6 ± 1.0	3.8 ± 0.9	NS
Heating at 56°C for 30 min		1.1 ± 0.0	1.4 ± 0.4	3.8 ± 1.1	3.6 ± 1.0	NS

Values are expressed as means ± standard error of the mean. Values with different superscript letters (e.g., a, b, and c) within the same row are significantly different (Tukey’s Honestly Significant Difference test, p < 0.05). “a” represents the highest mean value in each row. Groups that share the same letter are not significantly different. RBC = Red blood cells, SRBC = Sheep red blood cells

In yellow camels, the highest titers occurred against black RBCs, reaching 1338.4 ± 119.3 in >10-year-old animals. Heat inactivation or SRBC adsorption reduced titers drastically to as low as 0–16, indicating high sensitivity of yellow camel agglutinins to these treatments. Similar trends were observed when yellow serum was tested against white RBCs (115.5 ± 28.4 to 196.4 ± 38.9 untreated; markedly reduced post-treatment).

Black camel serum showed moderate titers against yellow or white RBCs (28.4 ± 6.7 to 112.6 ± 14.8), which decreased to 1.8 ± 0.6–14.4 ± 4.8 after SRBC adsorption and further to 2.0 ± 0.8–1.6 ± 2.9 after heating.

White camel serum displayed the lowest titers (115.5 ± 28.4–64.2 ± 14.9), with SRBC incubation having minimal impact (remaining around 3.8 ± 0.9), while heating reduced activity to 1.1 ± 0.0–3.6 ± 1.0.

Overall, non-specific agglutination was most pronounced in yellow camel serum, particularly against black RBCs, and was highly heat- and adsorption-sensitive. Black and white sera showed weaker and more treatment-resistant reactivity.

### Complement C3 concentrations by phenotype and age

**Figure 2:**
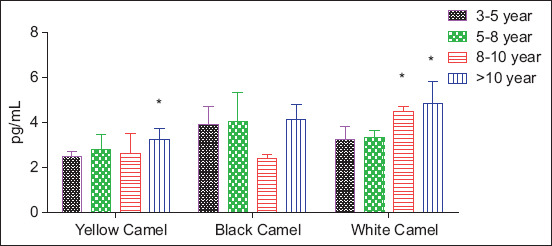
Camel C3 serum concentrations in different breeds at different ages. Columns marked with * are more significant compared with other ages within the same breed at p < 0.05.

Table 3 and Figure 2 illustrate phenotype- and age-associated variations in serum C3 levels. White camels exhibited a significant age-dependent increase in C3, from 3.252 ± 0.578 pg/mL (3–5 years) to 4.829 ± 0.983 pg/mL (>10 years; p < 0.05). Increases were particularly marked in the 8–10-year (4.482 ± 0.223 pg/mL) and >10-year groups. Yellow camels showed relatively stable C3 concentrations across most ages, except for a significant increase in the >10-year group (3.271 ± 0.482 pg/mL). Black camels displayed moderate C3 levels, with less pronounced age-related changes. These trends suggest phenotype-specific complement regulation and possible differences in immune system maturation or chronic activation across breeds.

**Table 3:** Camel C3 (pg/mL) in different breeds at different ages.

Age	Yellow Camel	Black Camel	White Camel
3–5 year	2.508 ± 0.220	3.937 ± 0.791	3.252 ± 0.578
5–8 year	2.802 ± 0.671	4.051 ± 1.264	3.318 ± 0.315
8–10 years	2.620 ± 0.906	2.430 ± 0.175	4.482 ± 0.223*
>10 year	3.271 ± 0.482*	4.150 ± 0.647	4.829 ± 0.983*

In the same column, values marked with

* are more significant than other ages at P < 0.05

### Complement C5a concentrations by phenotype and age

**Figure 3:**
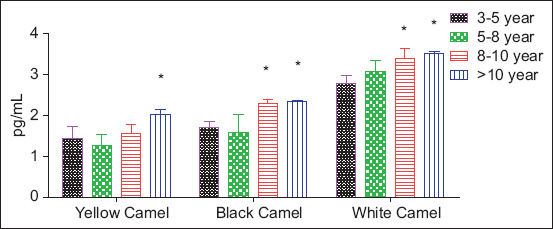
Camel C5a serum concentration in different breeds at different ages. Columns marked with * are more significant compared with other ages within the same breed at p < 0.05.

Table 4 and Figure 3 show significant age- and phenotype-dependent differences in C5a concentrations.

**Table 4:** Camel C5a (pg/mL) in the serum of different breeds with different ages.

Age	Yellow Camel	Black Camel	White Camel
3–5 year	1.446 ± 0.278	1.703 ± 0.153	2.776 ± 0.205
5–8 year	1.260 ± 0.269	1.577 ± 0.446	3.083 ± 0.264
8–10 years	1.561 ± 0.225	2.299 ± 0.104*	3.390 ± 0.258*
>10 year	2.018 ± 0.130*	2.347 ± 0.0204*	3.525 ± 0.052*

In the same column, values marked with

* are more significant than other ages at p < 0.05

White camels maintained the highest C5a levels across all age groups (2.776–3.525 pg/mL), with significant increases in the 8–10-year (3.390 ± 0.258 pg/mL) and >10-year (3.525 ± 0.052 pg/mL) groups.

Black camels had moderate baseline C5a, which rose significantly in the 8–10-year (2.299 ± 0.104 pg/mL) and >10-year (2.347 ± 0.0204 pg/mL) groups.

Yellow camels displayed the lowest baseline C5a but still showed a significant increase with age, reaching 2.018 ± 0.130 pg/mL in the >10-year group.

These results indicate that all three phenotypes undergo complement system activation with age, but the magnitude and baseline levels vary, with white camels showing the strongest constitutive complement activity, followed by black, and then yellow camels. This may reflect genetically determined differences in immune responsiveness or adaptation to environmental pressures.

## DISCUSSION

### Phenotype- and age-dependent variations in non-specific agglutinin reactivity

The findings reveal significant variations in non-specific agglutinin reactivity among camel phenotypes (yellow, black, and white), with age further modulating these responses. The robust agglutination activity of yellow camel serum, particularly against black and white red blood cells (RBCs), suggests intrinsic immunological differences linked to phenotypes. This observation aligns with prior research demonstrating that camelid antibodies, including natural agglutinins, exhibit unique structural and functional properties compared with those of other mammals [13].

The heightened reactivity observed in yellow camels may reflect evolutionary adaptations to pathogen exposure or environmental stressors, similar to other species where phenotypic traits correlate with immune function [14]. The age-dependent increase in agglutinin titers, especially in yellow camels, parallels observations in humans and other mammals, where immune maturation and cumulative antigen exposure enhance antibody production [15]. The rise in titers from 216.6 ± 58.4 (3–5 years) to 1338.4 ± 119.3 (>10 years) against black RBCs underscores the dynamic nature of camel immunity. Similar age-related trends have been documented in equine and bovine studies, where older animals exhibited stronger innate immune responses [15].

In contrast, black camel serum exhibits moderate agglutination titers, especially against RBCs from yellow camels, with dilution endpoints ranging from 28.4 ± 7.3 in younger camels to 196.4 ± 38.9 in older camels [15]. Agglutination reactions against white camel RBCs are weaker, varying from 28.4 ± 6.7 to 112.6 ± 14.8, indicative of selective antigenic recognition or limited immunogenic exposure in the black camel phenotype [15].

### Implications for diagnostics and disease testing

The non-specific agglutination observed in this study may complicate serological diagnostics, mirroring challenges reported in camelid infectious disease testing [16]. Pre-adsorption techniques, which have been validated for reducing false positives in ruminant sera [17], could be adapted for use in camels. Further research should characterize these agglutinins (e.g., IgM vs. IgG) and assess their role in hemolytic disease.

### Influence of serum treatments on agglutination titers

This study also demonstrates significant variability in non-specific agglutinin reactivity following serum treatments (heat inactivation and SRBC adsorption), both of which profoundly influenced agglutination titers. The pronounced reactivity of yellow camel serum, particularly against black camel RBCs, suggests the presence of high-affinity natural antibodies, possibly due to genetic or environmental factors driving stronger innate immune responses in this phenotype. Similar findings have been reported in other species, where natural antibodies play a crucial role in immune defense and cross-reactivity [18].

The drastic reduction in agglutination titers after heat treatment (56°C for 30 min) indicates that these agglutinins are likely heat-labile, a characteristic of IgM antibodies known for their sensitivity to thermal denaturation [19]. Camel serum proteins, particularly immunoglobulins such as IgG and albumin, exhibit notable heat stability, an adaptation crucial for survival in high-temperature desert environments [20]. Likewise, a study by Alvarez *et al*. [21] in humans has shown that selective inactivation of antibodies on heating leads to decreased agglutination titers and altered immunological reactivity.

The near-complete elimination of reactivity after SRBC adsorption further supports the hypothesis that these are polyreactive natural antibodies, as pre-adsorption with heterologous RBCs is a well-established method to remove non-specific agglutinins [21]. Black camel serum exhibited moderate reactivity, which was also significantly reduced by heat and adsorption, although to a lesser extent than that of yellow serum, suggesting a lower agglutinin concentration or avidity, possibly due to differences in immune regulation between phenotypes. Similar variations in natural antibody profiles have been observed in cattle, where breed-specific differences influence humoral immunity [22].

White camel serum displayed the weakest reactivity, with minimal reduction after SRBC adsorption but near-complete loss after heating. White camels may produce fewer natural agglutinins, or their antibodies may be less cross-reactive. The persistence of low titers after adsorption suggests residual non-IgM activity, possibly IgG, which is more heat-stable but less agglutinating [23].

### Transfusion risks and phenotype matching

The strong reactivity of yellow camel serum against black and white RBCs highlights the risk of hemolytic reactions in cross-phenotype transfusions, necessitating phenotype matching, as practiced in equine and canine blood banking [24]. The heat and adsorption sensitivities of these agglutinins suggest that sera pretreatment could reduce false positives in serological assays, similar to protocols used in bovine brucellosis testing [16]. Phenotype-dependent differences may reflect evolutionary adaptations, wherein yellow camels develop stronger natural antibody responses when exposed to broader pathogen pressures [14]. Given the robust agglutination observed in certain phenotypes, particularly yellow camels, meticulous blood cross-matching procedures are essential in veterinary transfusion practices [25].

### Complement system dynamics and age effects

The complement system, particularly C3 and C5a, plays a crucial role in immune responses during blood transfusion. The activation of the complement cascade can enhance phagocytosis, promote inflammation, and contribute to adverse transfusion outcomes [26].

#### C3 variations

The observed variations in complement C3 levels among different camel phenotypes and age groups provide valuable insights into complement system maturation and activation. White camels exhibit a significant age-dependent increase in C3, suggesting cumulative activation or maturation with age, possibly due to prolonged exposure to pathogens or intrinsic immunological changes [27]. Similar age-related increases in complement activity have been reported in other species, with older animals showing heightened inflammatory markers due to immunosenescence or chronic low-grade inflammation [28].

Yellow camels displayed relatively stable C3 concentrations across most age groups, except for the oldest cohort, which showed a significant increase. This delayed increase may indicate a slower or less pronounced complement activation compared with that in white camels. The phenotypic differences in C3 dynamics may be linked to genetic variations that influence immune regulation, as coat color-related genes have been associated with immune function in other mammals [29].

The most striking elevation in C3 was observed in white camels aged 8–10 and >10 years, suggesting enhanced complement activation in later stages of life. This may reflect an increased inflammatory state, possibly due to age-related immune dysregulation or higher susceptibility to infections [30]. Age significantly influences the levels of various blood constituents in camels, including proteins and immune factors [31]. Studies have shown that serum protein levels in dromedary camels increase with age, suggesting that complement components, such as C3, may also exhibit age-related variations. Elevated C3 has been linked to chronic inflammatory conditions in other livestock species [32]. Comparisons with Bactrian camels [33] and cattle [32] highlight that dromedaries exhibit higher baseline C3 than Bactrians, possibly due to arid adaptation.

#### C5a variations

White camels exhibited the highest C5a levels across all age groups, with a statistically significant increase in older animals. This progressive elevation suggests robust and sustained activation of the complement system with age, possibly due to enhanced innate immune responsiveness or genetic predisposition [34]. Similar breed-dependent immune variations have been observed in other livestock [30], where certain phenotypes exhibit stronger inflammatory responses.

Black camels exhibited moderate C5a levels, which became significantly elevated in older age groups, indicating a delayed but notable maturation of their complement system. This pattern is consistent with findings in other species where immune maturation occurs later in life [27].

Yellow camels displayed the lowest baseline C5a concentrations, although they still exhibited a significant age-dependent increase. The phenotypic differences in C5a levels could be linked to variations in key complement regulatory genes or environmental adaptations [32].

The progressive increase in C5a with age across all breeds may reflect cumulative immune exposure, chronic low-grade inflammation, or developmental shifts in complement protein synthesis [28]. The expression of cell adhesion molecules is reduced in older camels, which may impact immune cell migration and function [35]. Renal aging in camels, including declines in podocin expression and structural changes, may alter complement regulation and lead to higher C5a levels [36]. Breed-specific differences in mucosal immunity and immune cell composition, such as the higher neutrophil fraction in the Majaheem breed compared to the Magateer breed [36], may also affect complement activation.

The complement system in camels, as in other mammals, is activated through three pathways: The classical, lectin, and alternative pathways. These pathways lead to the generation of bioactive molecules, including C5a, which plays a central role in inflammation and immune defense [37]. The aging process camels may lead to increased complement activation, resulting in elevated C5a levels. This hypothesis is supported by studies showing that older camels exhibit higher levels of inflammatory mediators, such as proinflammatory cytokines, which are often associated with complement activation [38]. Studies on camel renal aging have shown significant declines in podocin expression and increased smoothening, which could affect the kidneys’ ability to regulate complement factors such as C5a [39], resulting in higher circulating C5a levels in older camels. Studies on Bactrian camels have shown age-related changes in IgA and IgG antibody-secreting cells distribution in mucosal tissues, which could influence local complement activity [39]. Breed-specific differences in mucosal immune responses may also affect complement activation and C5a expression. Camel breeds exhibit differences in immune cell composition and function. For instance, the Majaheem breed has a higher neutrophils fraction than the Magateer breed, which may indicate breed-specific variations in innate immune responses [39]. Such differences could extend to complement activity, potentially leading to variations in C5a levels between breeds [40, 41].

## CONCLUSION

This study demonstrates clear phenotype- and age-dependent variations in non-specific agglutinin reactivity and complement activation in dromedary camels. Yellow camels exhibited the highest agglutination titers, particularly against black and white RBCs, with age-associated increases from 216.6 ± 58.4 in 3–5-year-olds to 1338.4 ± 119.3 in camels over 10 years. Black camels showed moderate titers, and white camels displayed the weakest reactivity. Heat inactivation (56°C for 30 min) and SRBC adsorption significantly reduced agglutination, confirming the predominance of heat-labile, likely IgM-mediated activity. Complement analysis revealed that white camels had the highest C3 and C5a levels, particularly in older age groups, indicating sustained complement activation with aging. Yellow camels showed lower baseline C5a, whereas black camels displayed delayed increases.

These findings have direct implications for camel transfusion medicine and disease diagnostics. Phenotype-matched transfusions are strongly recommended, particularly avoiding yellow-to-black/white blood transfers to reduce hemolytic risk. Heat inactivation protocols could be integrated into pre-transfusion processing to mitigate IgM-mediated reactions. Complement profiling, especially C5a quantification, may serve as a predictive biomarker for inflammatory transfusion complications. The insights also inform cross-matching protocols, diagnostic assay refinement, and breeding strategies targeting enhanced immune compatibility.

The study benefits from a large sample size (n = 360), standardized agglutination and complement assays, and detailed phenotype-age stratification. The integration of both antibody reactivity and complement dynamics offers a comprehensive view of innate immune variation in camels, a topic with limited prior documentation. However, key limitations include the absence of cross-reactivity testing with RBCs from other species (e.g., bovine and equine), lack of genetic data (e.g., complement gene SNPs), and no direct differentiation between IgM and IgG subclasses through immunochemical assays. These factors may limit the generalizability and mechanistic interpretation of the findings.

Further research should involve GWAS to identify loci influencing agglutinin and complement activity, coupled with functional immunophenotyping to confirm antibody class involvement. Studies on environmental and pathogen exposure histories could clarify the ecological drivers of phenotype-specific immune responses. Expanded cross-matching trials across diverse livestock species will enhance transfusion safety guidelines. Overall, this study provides novel, phenotype-specific immunological insights that advance our understanding of camelid innate immunity. The evidence strongly supports the need for phenotype-aware transfusion protocols, heat treatment of donor sera, and complement monitoring to prevent adverse reactions. These measures, alongside genetic and immunological profiling, will enhance clinical safety, improve diagnostic accuracy, and contribute to optimized health management in camel populations.

## DATA AVAILABILITY

The supplementary data can be available from the corresponding author upon request.

## AUTHOR’S CONTRIBUTIONS

YMA: Study design, methodology, and drafted and revised the manuscript. The author has read and approved the final manuscript.
